# The longitudinal assessment of prenatal cannabis use on neonatal outcomes

**DOI:** 10.1038/s41372-024-02027-w

**Published:** 2024-06-18

**Authors:** Leah L. Habersham, Yasmin L. Hurd, Yoko Nomura

**Affiliations:** 1 Department of Psychiatry, Icahn School of Medicine at Mount Sinai, New York, NY US; 2 Department of Obstetrics and Gynecology, Icahn School of Medicine at Mount Sinai, New York, NY US; 3 Addiction Institute of Mount Sinai, New York, NY US; 4 Queens College and Graduate Center, The City University of New York, New York, NY US

**Keywords:** Addiction, Outcomes research

## Abstract

**Objective:**

To investigate the association between prenatal cannabis use and perinatal outcomes using longitudinal data from pregnant individuals.

**Study design:**

This secondary-data analysis study utilized data collected from 894 pregnant individuals followed in the Stress in Pregnancy longitudinal study, conducted between 2009 and 2013. The status of cannabis use was ascertained through interviews and electronic medical record reviews to evaluate the effect of cannabis use on perinatal outcomes (NICU admission, preterm delivery, low birth weight, fetal death).

**Results:**

Among participants analyzed, 13.1% used cannabis, who were generally younger (25.9 vs 27.9 years). There was a sevenfold increased risk of fetal death (OR 7.30) among cannabis users relative to non-users. Elevated risk persisted after adjustments of potential confounders (aOR 6.31). Adjusted models also suggested increased low birth weight risk (aOR 1.67).

**Conclusion:**

This study highlights an association between prenatal cannabis use and elevated risks for fetal death and low birth weight.

## Introduction

With the evolving paradigm shift towards cannabis decriminalization and legalization, the acceptance of recreational cannabis has steadily risen throughout the United States. This acceptance is reflected in the increasing use of cannabis evident in many demographic groups including pregnant individuals. Based on data from the National Survey on Drug Use & Health, ~21.6% of pregnant women did not view weekly cannabis use to carry any risk [1]. Moreover, cannabis is commonly perceived as beneficial to reduce nausea during pregnancy without any health risk to the offspring [1]. This perception stands in contrast to the current body of literature that suggests potential negative impacts of cannabis use during pregnancy on fetal outcomes [2–15].

There is well-accepted data demonstrating the risk of low birth weight of infants, a well-known predictor for infant morbidity and mortality, born to individuals who used cannabis during pregnancy [2–4]. Other adverse birth outcomes, such as preterm birth, intrauterine growth restriction, neonatal intensive care unit (NICU) admission, developmental delays, smaller head circumference, and neurobehavioral issues, are often correlated with low birth weight and have also been associated with prenatal cannabis use [2–5, 7, 8, 10, 12]. However, inconsistent and limited results exist regarding the relationship between prenatal cannabis use and perinatal loss such as spontaneous abortion or stillbirth related to maternal substance use [5–8, 11, 12]. Prior evidence has shown that chronic cannabis use can adversely influence implantation and placentation development, through disruption of local endocannabinoid processes [9]. Given the critical role of the endocannabinoid system in feto-placental development, exposure to cannabis and disruption of the endocannabinoid system could lead to complications of early pregnancy such as ectopic pregnancy and spontaneous abortion [9]. In the current study, we evaluated the association of cannabis use in pregnancy with neonatal outcomes. This study adds to the current literature through its longitudinal evaluation of patients during pregnancy.

## Materials/subjects and methods

**Fig. 1:**
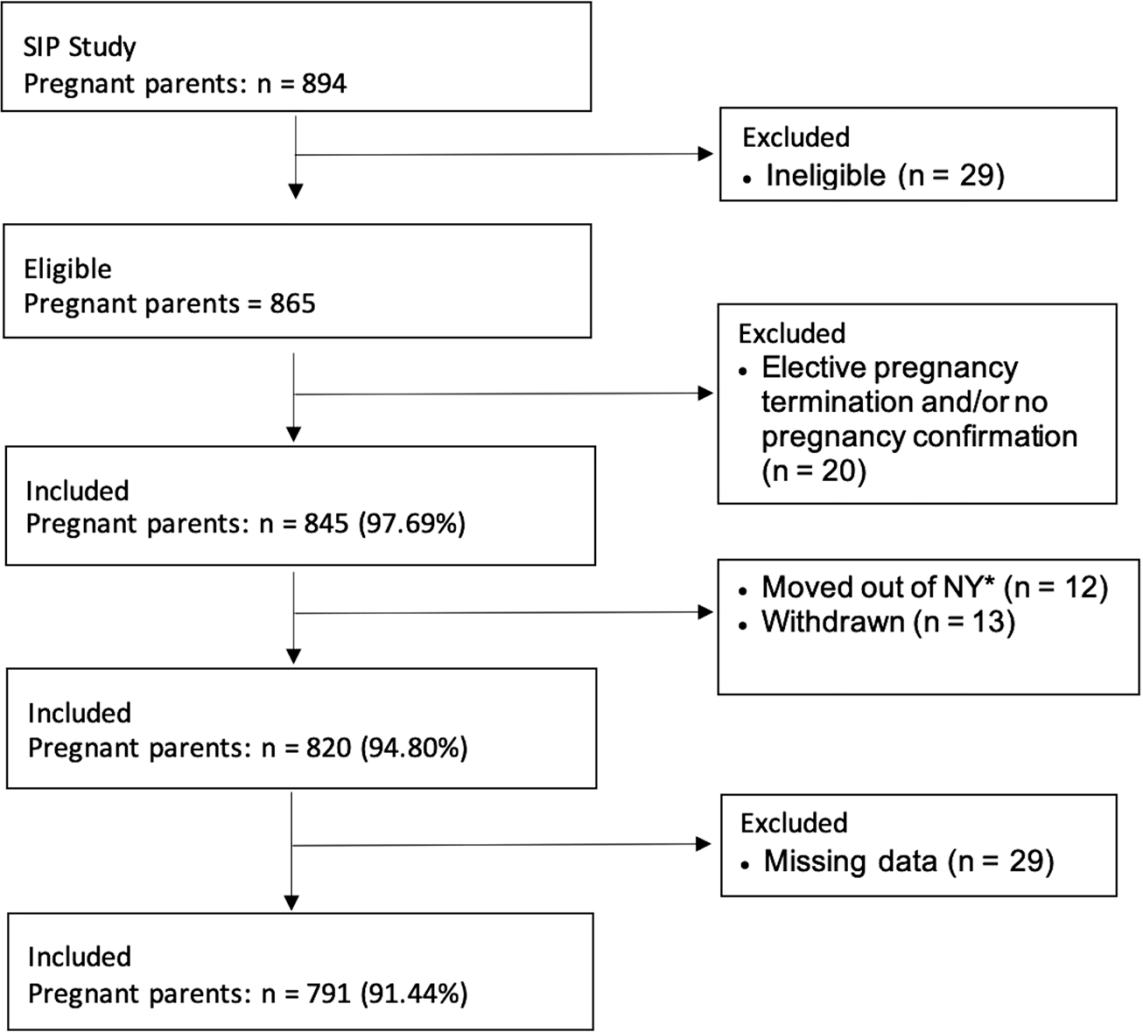
Flowchart of participants included in the study. *NY New York.

This was a retrospective cohort study, performed leveraging data collected from the Stress in Pregnancy (SIP) longitudinal study, evaluating the association between cannabis use and several neonatal outcomes, including fetal death (defined as any one of the following: spontaneous abortion, fetal demise *in utero*, and stillbirth) [16], low birthweight, preterm delivery, and NICU admission. The SIP study was carried out at two prenatal obstetric clinics in metropolitan New York on pregnant individuals between 2009 and 2013 [17]. Data was collected directly from interviews of 894 participants as well as from review of their electronic medical records. Informed consent was obtained for each participant. Participants were excluded if they had maternal or fetal risk factors including HIV, congenital anomalies, inborn errors of metabolism, if they had plans to relocate out of the geographic area, or if they moved out of the geographic area (see Fig. 1) [17]. This study was approved by the City University of New York (CUNY) Institutional Review Board (protocol #2023-0369).

We evaluated several variables, including maternal age, race, maternal weight, marital status, education, prior spontaneous abortion, nicotine use, and cannabis use. Among these, cannabis use was the primary predictor variable of interest. Maternal age at the time of initial entry into the study was recorded. Race and ethnicity were identified as either Asian, Black, Hispanic, Other, or White. Other designation was used when a participant identified as multi-racial. Participants pre-pregnancy weight was based on the self-report upon entry to the study. Substance use was determined through participant clinical interviews. The substances assessed included nicotine, cannabis, alcohol, cocaine, benzodiazepines, opioids, stimulants, hallucinogens, and polysubstance use. Those participants who denied use of cannabis or other substances, and whose chart review demonstrated no evidence of substance use were designated as nonusers of the corresponding substance.

Several perinatal outcomes were evaluated, including NICU admission, preterm delivery, low birth weight all noted at the time of birth, while fetal death was assessed longitudinally across the prenatal period.

Descriptive statistics were first conducted using *t*-tests for continuous measures and Chi-squared tests for categorical data. The primary predictor of interest was cannabis use. Covariates were categorized into two groups: modifiable factors, representing characteristics individuals can alter, and non-modifiable factors, which are typically unchangeable characteristics. Modifiable covariates include nicotine use and marital status. Non-modifiable covariates include maternal age and race. To assess the relationship between cannabis use (the primary predictor) and neonatal outcomes, we analyzed unadjusted odds ratios (OR) and adjusted odds ratios (aOR) with their corresponding 95% confidence intervals (95%CI). Adjustments were made for both modifiable and non-modifiable covariates in two blocks using logistic regression. All analyses were conducted using SAS OnDemand for Academics (SAS, Cary, NC, USA).

## Results

A total of 791 pregnant individuals met the inclusion and exclusion criteria without missing data on cannabis use during pregnancy. The only substances reported to be used during pregnancy within this cohort were cannabis, nicotine, and alcohol. Table 1 provides a detailed breakdown of the baseline demographic characteristics of the study population. There were pronounced differences between cannabis users and non-users. The mean age for cannabis users was younger, at 25.9 (SD 4.93) years, in comparison to non-users with an average age of 27.9 (SD 5.82) years (*P* = 0.0003). In terms of racial distributions, Black participants were overrepresented among cannabis users (37.5%) than non-users (22.7%). In contrast, Asian participants were underrepresented among cannabis users (1.9%) compared to non-users (9.0%) (*P* = 0.0006). Approximately half of the participants identified as Hispanic, with similar proportions between cannabis users and non-users. Notably, a significant majority of unmarried individuals, 85.6%, reported cannabis use, contrasting sharply with the 14.4% among those married (*P* < 0.0001). Cannabis users also reported a higher prevalence of nicotine use, 17.5%, compared to 8.7% in non-users (*P* = 0.01).

**Table 1:** Demographic and social characteristics of prenatal participants according to cannabis use.

	Total	No prenatal cannabis use (*N* = 687)	Prenatal cannabis use (*N* = 104)	*P-*value
	*N* (Mean)	Mean (SD)	Mean (SD)	
Age (years)	791 (27.65)	27.91 (5.82)	25.94 (4.93)	0.0003
Parity	721 (1.97)	2.02 (1.79)	1.69 (1.57)	0.09
Race	*N* (%)	*N* (%)	*N* (%)	0.0006
Asian	64 (8.1)	62 (9.0)	2 (1.9)	
Black	195 (24.7)	156 (22.7)	39 (37.5)	
Hispanic	398 (50.3)	345(50.2)	53 (50.9)	
Other	33 (4.2)	28 (4.1)	5 (4.8)	
White	101 (12.8)	96 (13.9)	5 (4.8)	
Education^a^		*N* (%)	*N* (%)	0.19
≥HS	139 (17.6)	116 (16.9)	23 (22.1)	
<HS	652 (82.4)	571 (83.1)	81 (77.9)	
Marital status		*N* (%)	*N* (%)	<0.0001
Married	284 (35.9)	269 (39.2)	15 (14.4)	
Unmarried	507 (64.1)	418 (60.8)	89 (85.6)	
Prior spontaneous abortion		*N* (%)	*N* (%)	0.71
No	447 (56.5)	381 (74.4)	66 (72.5)	
Yes	156 (19.7)	131 (25.6)	25 (27.5)	
Nicotine use^b^		*N* (%)	*N* (%)	0.01
No	642 (81.2)	557 (91.3)	85 (82.5)	
Yes	71 (9.0)	53 (8.7)	18 (17.5)	

^a^HS, High school, ≥HS is completion of education level of high school or greater; <HS is less than completion of education level of high school.

^b^Nicotine use during pregnancy.

When examining neonatal outcomes, prenatal cannabis use presented varied consequences. Unadjusted models (Table 2) did not indicate a significant elevation in risks tied to maternal cannabis use for factors such as low birth weight (OR 1.51, 95%CI [0.99 to 2.31]), NICU admission (OR 0.73, 95% CI [0.28 to 1.89]), or preterm birth (OR 0.73, 95% CI [0.32 to 1.65]). Yet, after adjustments were made for various risk factors in the model (Table 2), a marked increase in risk for low birth weight became evident (aOR 1.67, 95%CI [1.06 to 2.62]). Risks associated with preterm birth (OR 0.63, 95% CI [0.29 to 1.60]) or NICU admission (OR 0.63, 95% CI [0.24 to 1.68]) did not reach a level of significance. A striking finding was the over sevenfold increase in risk of fetal death linked to maternal cannabis use in the unadjusted model (OR 7.30, 95% CI [3.08 to 17.30]). This elevated risk persisted, with significance (aOR 6.31, 95% CI [2.47 to 16.17]), even after accounting for variables such as maternal nicotine use, marital status, age, and race. Alcohol use showed no association with neonatal outcomes.

**Table 2:** Prevalence (per 100) and risk (odds ratio) of suboptimal perinatal outcomes by prenatal cannabis use.

Neonatal outcomes	Prenatal cannabis use		Unadjusted model	Adjusted^a^ model
	Yes *N* (%)	No *N* (%)	OR (95% CI) *p*-value	aOR (95% CI) *p*-value
Fetal death	11 (1.6%)	11 (10.7%)	7.30 (3.08–17.30) <0.00001	6.31 (2.47–16.17) 0.0001
Low birthweight (<2500 g)	42 (40.8%)	214 (31.3%)	1.51 (0.99–2.31) 0.06	1.67 (1.06–2.62) 0.03
Preterm birth (<37 weeks)	7 (9.5%)	66 (12.6%)	0.73 (0.32–1.65) 0.45	0.63 (0.29–1.60) 0.38
NICU admission	5 (4.9%)	43 (6.7%)	0.73 (0.28–1.89) 0.52	0.63 (0.24–1.68) 0.35

*OR* odd ratio, *aOR* adjusted odd ratio, *NICU* neonatal intensive care unit.

^a^Race, age, parity, nicotine use, education, and marital status are statistically adjusted for in the model.

## Discussion

This study was conducted to evaluate whether an association exists between cannabis use during pregnancy and neonatal outcomes given the growing acceptance and subsequent use of cannabis, particularly amongst pregnant individuals and those of childbearing age. Based on adjusted findings, our results reaffirm the association of cannabis use during pregnancy with significant adverse neonatal outcomes, specifically a more than sixfold elevated risk for fetal death and nearly a twofold increased risk for low birth weight.

The association between cannabis use and fetal death observed in our study is consistent with some of the existing literature suggesting detrimental effects of cannabis on fetal outcomes [5, 6, 12]. Even after adjusting for modifiable (nicotine use, marital status) and non-modifiable covariates (maternal age and race), the risk associated with cannabis use remained markedly elevated. These findings may relate to the integral role of endocannabinoids and endocannabinoid signaling in the modulation of gestational events. For example, the tight regulation of the endocannabinoid ligand anandamide (AEA) is known to be necessary for successful embryo implantation [9]. Moreover, when levels of AEA are impaired, a spontaneous abortion or ectopic pregnancy may result [9, 18–21]. AEA can also have disruptive effects on decidualization, which is important for normal embryo and placental development [9, 22]. AEA mediates its actions at cannabinoid receptors the same target for Δ^9^ tetrahydrocannabinol (THC), the main psychoactive component of cannabis, which is known to disrupt AEA levels [9]. Our findings would thus be in line with the crucial role that the endocannabinoid system plays in normal gestational development.

Cannabis use during pregnancy has often been shown to be associated with an increased risk for low birth weight [2, 4, 6, 13–15]. Although we did not find a significant increase in the risk of low birth weight, preterm birth, or NICU admission in our unadjusted models evaluating cannabis use, there was a significant risk for low birth weight when cannabis use was factored in along with other predictor variables. Therefore, this finding indicates that there may be a more complex relationship between cannabis use during pregnancy and low birth weight. Given the strong association between low birth weight on offspring morbidity, these results may have implications for not only fetal but also subsequent infant/early child health and development.

Contrary to reported associations in the literature, our analysis did not find alcohol use during pregnancy to be associated with adverse effects [23–25]. Our findings may reflect differences in patterns of use or population characteristics, leading to these unanticipated results.

While our study provides valuable insights into the impact of prenatal cannabis use on neonatal outcomes, we recognize its limitations. First, characterization of substance use is lacking, without detailed description of substance mode, exact timing (including gestation), duration, or amount. Second, our data does not include toxicology testing to serve as corroboration of interview-derived substance use history. Third, our findings are limited to pregnant individuals seeking prenatal care at urban settings thus restricting the generalizability. Despite these limitations, our study did replicate other findings in the literature and a notable strength of our study is the longitudinal nature of the data.

In summary, our findings highlight a significant association between prenatal cannabis use and adverse neonatal outcomes, i.e., fetal death and low birth weight. The noted association between fetal death and cannabis use during pregnancy emphasizes the need for larger studies that evaluate individuals’ substance use throughout the gestational period, beginning either pre-pregnancy or early pregnancy, to better determine whether there may be particularly sensitive windows in fetal development. In addition, identifying potential mechanistic underpinnings that specifically link cannabis use with fetal death would be important. Overall, our findings illuminate the need for interventions aimed at educating individuals of childbearing age about the potential risks associated with cannabis use during pregnancy. Given the increasing acceptance and use of cannabis, along with greater THC potency [26], it is crucial to expand research in this field to ensure the health and safety of both pregnant parents and neonates.
